# Neural underpinnings of contingency awareness in human fear conditioning

**DOI:** 10.1192/j.eurpsy.2021.1298

**Published:** 2021-08-13

**Authors:** Y. Pavlov, B. Kotchoubey

**Affiliations:** 1 Department Of Psychology, Ural Federal University, Ekaterinburg, Russian Federation; 2 Institute Of Medical Psychology, University of Tuebingen, Tuebingen, Germany

**Keywords:** EEG, ERP, Fear conditioning, contingency awareness

## Abstract

**Introduction:**

The recognition of the conditioned-unconditioned stimulus (CS-US) association in classical conditioning is referred to as contingency awareness. The neural underpinnings of contingency awareness in human fear conditioning are poorly understood.

**Objectives:**

We aimed to explore the EEG correlates of contingency awareness.

**Methods:**

Here, we recorded electroencephalography (EEG) from a sample of 20 participants in a semantic conditioning experiment. In the acquisition phase the participants were presented with sequences of words from two semantic categories paired with tactile stimulation followed by presentation of a neutral sound (US-) ((e.g., animals -> left hand vibration -> US-, clothes -> right hand vibration -> US-). In the test phase the association violated in 50% of trials which followed by a presentation of a loud noise (US+). The participants were only instructed to listen carefully. On the basis of self-reported contingency awareness, twenty participants were divided in aware (N=12) and unaware (N=8) group.

**Results:**

The aware group expressed a non-lateralized effect of alpha-beta (12-23 Hz) suppression along with a more negative CNV at central channels preceding presentation of the vibration (main effect of Group). Also, CNV was more negative in expectation of US+ comparing with expectation of US- in the aware group but not in the unaware group.

**Conclusions:**

The results indicate that contingency awareness is accompanied by neural patterns reflecting expectation as can be seen in the suppression of somatosensory alpha-beta activity before expected presentation of the vibration as well as in CNV in expectation of an aversive event.

